# Cells Expressing the C/EBPbeta Isoform, LIP, Engulf Their Neighbors

**DOI:** 10.1371/journal.pone.0041807

**Published:** 2012-07-31

**Authors:** Maria Abreu, Linda Sealy

**Affiliations:** 1 Department of Cancer Biology, Vanderbilt University School of Medicine, Nashville, Tennessee, United States of America; 2 Department of Molecular Physiology and Biophysics, Vanderbilt University School of Medicine, Nashville, Tennessee, United States of America; Chinese University of Hong Kong, Hong Kong

## Abstract

Descriptions of various processes that lead to cell-in-cell structures have been reported for decades. The exact molecular mechanism(s) of their formation and the physiological significance of cell-in-cell structures remain poorly understood. We had previously shown that an isoform of the CCAAT/enhancer-binding protein beta (C/EBPbeta) transcription factor, liver-enriched inhibitory protein (LIP), induces cell death in human breast cancer cells and stimulates autophagy. Here we describe a non-apoptotic cell death process where LIP mediates the engulfment of neighboring cells. We provide evidence of LIP-mediated engulfment via DNA profiling, fluorescent imaging and cell sorting studies, as well as ultrastructure analysis of LIP-expressing MDA-MB-468 breast cancer cells. Our work illustrates that expression of a specific transcription factor, LIP, can mediate cell engulfment.

## Introduction

Recently there has been a revival of interest in the phenomenon of live cell engulfment or cell-in-cell structures, catalyzed in part by the description of a nonapoptotic cell death process, termed entosis, by Overholtzer et al [1]. Entosis occurs in matrix-detached cells, where viable target cells invade into viable host cells, forming cell-in-cell structures. However, reports of cell-in-cell structures date back to the mid 1800’s [2]. Many terms have been used in the literature to describe cell-in-cell structures including entosis, emperipolesis, cytophagocytosis, and cannibalism (xeno-cannibalism). Humble et al. were the first to introduce the term emperipolesis in the 1950’s to refer to a heterogeneous cell-in-cell phenomenon in which viable lymphocytes move into malignant cells [3]. During this process, the nucleus of the host cell is pressed to one side, and the internalized cell is housed in a large vacuole [3]. It has been proposed that emperipolesis denotes the process of cells entering, moving within, as well as exiting the cell, whereas cytophagocytosis, cannibalism, and entosis describe the specific mechanism of cell-in-cell formation [2]. While there are some overlapping similarities among the various mechanisms, entosis is a mechanism whereby target cells invade the host cell [1]. Conversely, in cell cannibalism a host cell actively engulfs the target cell. The ability of cannibal tumor cells to engulf other tumor cells resembles autophagic digestion of cellular organelles. For a review on different processes that lead to cell-in-cell structures please see [2].

Cell cannibalism has been frequently detected in highly malignant or metastatic tumors and has been correlated with poor prognosis [4]. This could possibly be due to the tumor cell’s ability to ingest immune cells such as lymphocytes and neutrophils for immune evasion [2], [4]. In contrast, natural killer (NK) cell internalization has been shown to precede target tumor cell death and NK cell self-destruction, suggesting that this cell-in-cell pathway is a mechanism to kill tumor cells [5]. This potential tumor suppressive function is similar to that observed in soft agar assays during entosis [1]. Nevertheless, the significance of cell-in-cell structures and the underlying mechanism(s) of their formation remain unknown.

Recently we described the non-apoptotic cell death of breast tumor cells upon the exogenous expression of LIP, an isoform of the C/EBPbeta transcription factor [6]. Transcription of the C/EBPbeta gene results in the expression of a single mRNA product that can generate three C/EBPbeta isoforms by alternative translation initiation at three in frame methionines [7]. The two larger isoforms C/EBPbeta-1 and -2 (also termed LAP* and LAP) are transcriptional activators, and only differ by 23 amino acids present in the N-terminus of C/EBPbeta-1. C/EBPbeta-3 (LIP) lacks the transactivation domain yet retains the DNA binding/protein dimerization domain and generally represses transcription [7], [8]. We have documented that high levels of LIP expression lead to the induction of autophagy and cell death in breast cancer cell lines such as MDA-MB-231 and MDA-MB-468 [6]. Here, we show that the induction of autophagy appears to accompany or possibly follow the engulfment of neighboring cells by the LIP-expressing cells. In 2–3 days up to 30–40% of LIP-expressing MDA-MB-468 cells have engulfed live cells, leading to extensive cell death. This study demonstrates that expression of a specific transcription factor can mediate cell engulfment.

## Results

### Cell Disintegration Following Exogenous Expression of LIP

**Figure 1:**
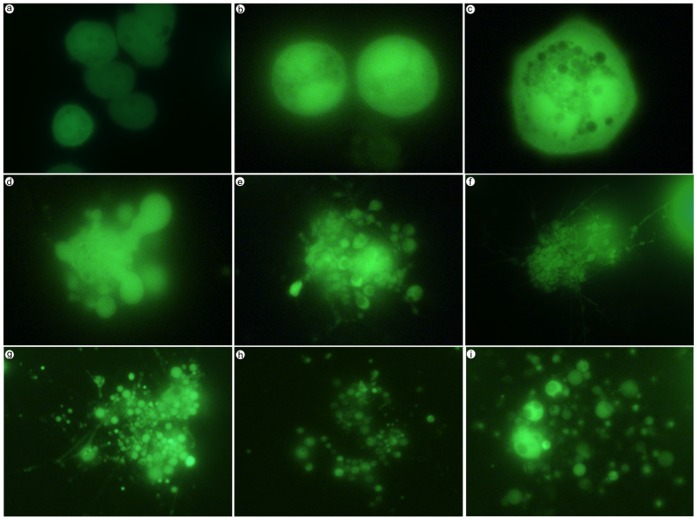
Cell disintegration following exogenous expression of LIP. Microscopic examination of cell breakdown. Equal numbers of MDA-MB-468 cells were plated for adenoviral infection with either control Ad-GFP or Ad-LIP. Representative fluorescent photomicrographs of cells imaged at 72 hrs post infection are shown. Panel a represents Ad-GFP MDA-MB-468 cells. Panels b-i represent Ad-LIP MDA-MB-468 cells.

Recently we reported a role for LIP in stimulating autophagy and causing cell death in breast cancer cell lines [6]. We evaluated the role of LIP overexpression in proliferation, necrosis, and LC3 protein turnover of MDA-MB-468 cells in particular. Exogenous expression of LIP in MDA-MB-468 cells leads to attenuation of cell proliferation as determined by cell growth assays and MTS assays. Colony formation assays show a dramatic reduction in colonies formed by LIP-expressing MDA-MB-468 cells [6]. This suggests overexpression of LIP causes cell death. In order to further characterize the mechanism of cell death due to LIP overexpression, we used an adenoviral (Ad) vector, Ad-LIP, to transduce the breast cancer cell line, MDA-MB-468, as previously described [6], [9]. Photomicrographs of MDA-MB-468 cells in culture 3 days post infection are shown in Figure 1. Images were taken at the same time point for all control Ad-GFP and Ad-LIP cells. A representative image of the control Ad-GFP MDA-MB-468 cells is shown in Figure 1, panel a. The remaining panels (b–i) capture the heterogeneity we observed among Ad-LIP infected cells. Some cells appear intact (Figure 1, panel b), while others appear very vacuolated (Figure 1, panel c). In addition, we observe a high percent of LIP-expressing cells breaking down into a number of smaller vesicles (Figure 1, panels d–i). Some of the vesicles appear to be held together by a network of fibers (Figure 1, panel f, g). These LIP-expressing cells will continue to disintegrate until eventually there is only cell debris in the culture medium.

### DNA Content of LIP-expressing MDA-MB-468 Cells

**Figure 2:**
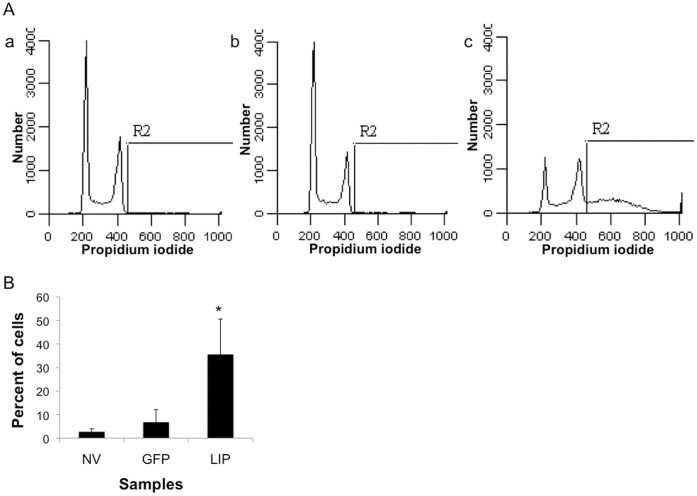
DNA content of LIP-expressing MDA-MB-468 cells. A, Representative cell cycle profiles of control uninfected: no virus (NV) (panel a), Ad-GFP (panel b), and Ad-LIP (panel c) MDA-MB-468 cells are shown. B, Quantification of the percent of cells with greater than 4n DNA content as measured by the area marked as R2 in the graphs is presented. Data shown represents the mean + SD of three separate experiments. *p-value <0.05 as determined by ANOVA followed by Friedman test.

We had observed that the extent of cell death was most severe in the MDA-MB-468 breast cancer cell line in comparison to other breast cancer cell lines tested [6]. To assess whether the dying cells were undergoing apoptosis we performed cell cycle profiling on the MDA-MB-468 cells expressing LIP. A sub-G1 phase characteristic of apoptotic cell death was not detected; even though we have shown above that cells are disintegrating and there is a clear breakdown into vesicles. Instead, we were surprised to find that LIP-expressing cells (Figure 2A panel c) have a significantly greater DNA content than the control uninfected and Ad-GFP MDA-MB-468 cells (Figure 2A panel a and b, respectively). Significant portions (>35%) of the LIP-expressing MDA-MB-468 cells have more than 4n DNA content at 72 hrs post infection (Figure 2B).

### LIP-expressing MDA-MB-468 Cells Engulf Neighboring Cells

**Figure 3:**
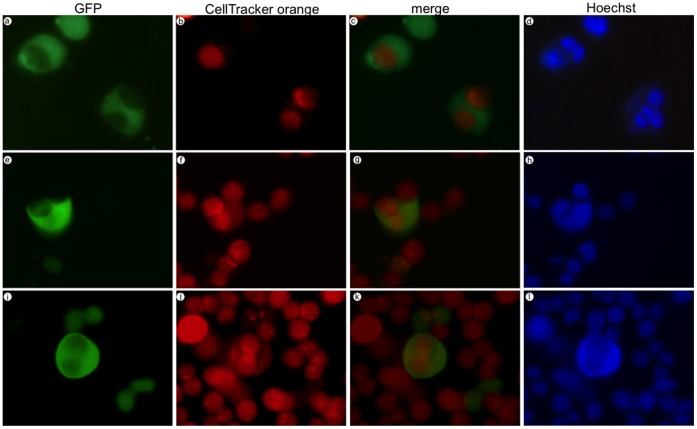
LIP-expressing MDA-MB-468 cells engulf neighboring cells. GFP positive LIP-expressing MDA-MB-468 cells were mixed with uninfected CellTracker orange labeled MDA-MB-468 cells. Hoechst dye was used to stain DNA. Cells were imaged at 36–48 hrs post-mix.

Over several days of examining LIP-expressing cells via microscopy, we noticed cells that appeared to be multinucleated as well as cells residing in large vacuoles. Taking this data together with the increase in DNA content, we reasoned it was possible for LIP overexpression to stimulate engulfment of neighboring cells. To further investigate this possibility, we took GFP positive LIP-expressing MDA-MB-468 cells and mixed them with a population of control uninfected MDA-MB-468 cells that were labeled with fluorescent CellTracker orange dye. After mixing the populations in a 1∶3 ratio, cells were plated and observed for 24–48 hrs. Engulfment of neighboring cells was evident beginning at 36 hrs post-mixing. We acquired fluorescent images of LIP-expressing cells (green) that had engulfed anywhere from one to three orange-labeled cells (Figure 3). DNA staining shows the appearance of multinucleated cells. Similar experiments were performed with control Ad-GFP MDA-MB-468 cells and this engulfment process was rare and occurred at a very low frequency.

To study engulfment in real time, GFP positive LIP-expressing MDA-MB-468 cells mixed with uninfected CellTracker-labeled MDA-MB-468 cells were analyzed by time-lapse microscopy over a 6 to 14 hr period. In work published by Overholtzer et al. they describe the entosis process to involve the active invasion of one cell into another [1]. As presented in Movie S1, we see the GFP positive LIP-expressing MDA-MB-468 cell extending its cell membrane and wrapping around the orange-labeled cell. These cells seem to battle for quite some time, with the orange cell trying to come out of the GFP positive LIP-expressing cell. However, the orange cell is not able to escape, at least within the time course of this movie (approximately 12 hrs). In Movie S2, there are a few green cells in the field that have orange-labeled cells inside. The GFP positive LIP-expressing cells are very active making contacts with neighboring cells and forming smaller vesicle-like structures. During this process, a group of GFP positive LIP-expressing cells come into view that have internalized orange-labeled cells. After some time, one of these cells begins to disintegrate into smaller vesicles until it eventually fades from the field. Interestingly, we were also able to capture a GFP positive LIP-expressing cell releasing an internalized orange-labeled cell. This is clearly seen in Movie S3 as the GFP positive LIP-expressing cell opens and the orange-labeled cell escapes or is expelled. It is difficult to conclude the fate of both of these cells, because they appear to detach and disappear from the plane of focus. In Movie S4, the GFP positive LIP-expressing cell clearly has engulfed more than one orange-labeled cell. One of the labeled cells is able to escape; however after the LIP-expressing cell rearranges, we see the LIP-expressing cell begin to breakdown and take the innermost-labeled cell with it. Both appear to disintegrate and again detach from the plane of focus.

It should be noted that GFP positive LIP-expressing cells will also engulf other GFP positive LIP-expressing cells as well. While we focus on the green cells engulfing the orange-labeled cells, this is not meant to imply that there is any preference for engulfing labeled orange cells; it is just easier to observe this photographically. Interestingly, we have not observed any GFP positive LIP-expressing cells inside of control uninfected orange-labeled cells. The GFP positive LIP-expressing cells do not invade the orange-labeled cells, as would be expected if the LIP-expressing cells were undergoing entosis.

### Ultrastructure Analysis of LIP-mediated Cell Engulfment

**Figure 4:**
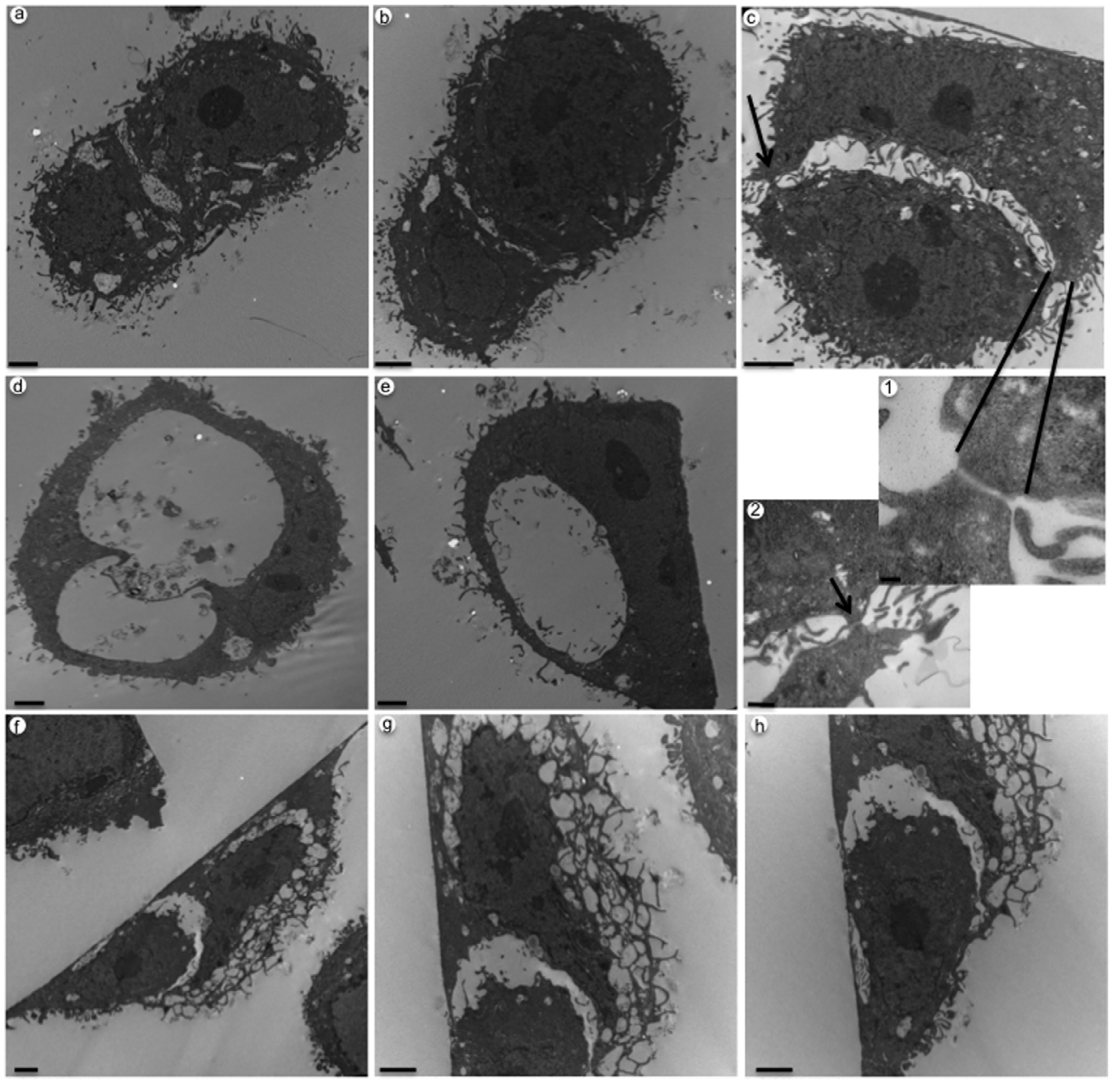
Ultrastructure analysis of LIP-mediated cell engulfment in MDA-MB-468 cells. Panels a–c, ultrastructure of engulfing intermediates at 48 hrs post infection; scale bar is 2µm. 1 and 2 indicate higher magnification of cell contacts present in panel c. Arrows indicate formation of cell contacts. Scale bar is 100 nm and 500 nm for panel 1 and 2, respectively. Panels d and e show LIP-expressing cells that have enormous vacuoles; scale bar is 2 µm. Panels f–h illustrate an internalized cell at 72 hrs post infection; scale bar is 2 µm. Panel g is a higher magnification to show the nucleus of engulfing (host) cell and the various projections typical of actively phagocytic cells. Panel h depicts the engulfed cell inside of the vacuole of the host cell.

To gain further confirmation of cell engulfment, transmission electron microscopy (TEM) analysis was performed at 48 and 72 hrs post infection of MDA-MB-468 cells with Ad-LIP. At 48 hrs we detect LIP-expressing cells extending their membranes and wrapping around the edges of neighboring cells (Figure 4 panels a–c). These engulfing intermediate cells appear to be vacuolated and forming cell contacts with the neighboring cells. It is unclear whether adherens junctions are being formed; yet fibers and secreted basement membrane can be detected (Figure 4 panels 1–2). Remarkably, the engulfing cell’s nucleus begins to change into a more crescent-like shape, characteristic of cannibalistic cells [4]. Figure 4, panels d and e, are examples of LIP-expressing cells that present with immense vacuoles. A captured cell that was able to escape may have previously occupied these large vacuoles, possibly leaving traces behind, or they could represent intake of extracellular fluid. In Figure 4, panels f–h, it is evident that a target cell has been internalized and resides in the large vacuole of the host cell. In these images we are able to detect more than one nucleus (Figure 4 panel f). Figure 4 panels g and h are magnified images that show the nuclei of both cells remain intact and the various projections typical of actively phagocytic cells. We had previously published work that LIP can stimulate autophagy in these cells [6]. Therefore, it is reasonable to see the presence of highly vacuolated cells as those seen in Figure 4 panels d–h. Taken together, these images provide strong evidence that LIP expression leads to the engulfment of neighboring cells in the MDA-MB-468 breast cancer cell line.

### Quantitation of LIP-mediated Cell Engulfment

**Figure 5:**
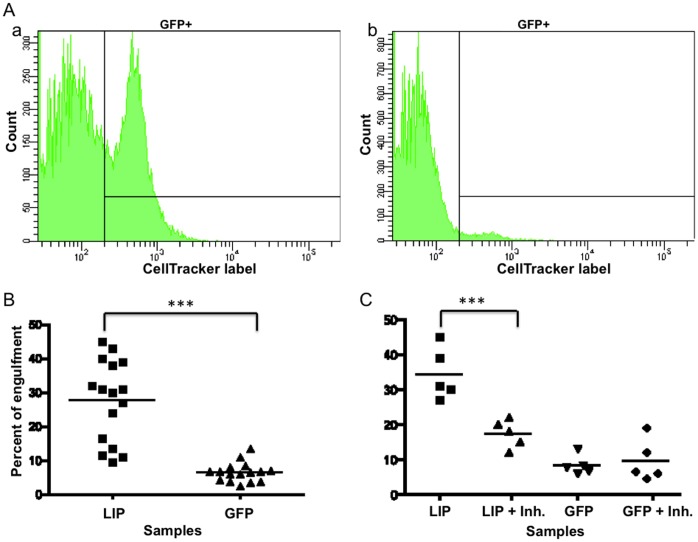
Quantification of LIP-mediated cell engulfment. A, Panel a shows the percent of Ad-LIP MDA-MB-468 cells that are double positive for GFP and CellTracker label. Panel b shows the percent of control Ad-GFP MDA-MB-468 cells that are double positive for GFP and CellTracker label. B, Quantification of the percent of GFP positive cells that have engulfed uninfected CellTracker labeled cells. Results are shown for 16 different experiments with a p-value of <0.0001 using paired t-test and Wilcoxon matched pairs test for statistical analysis. C, Quantification of the percent of GFP positive cells that have engulfed uninfected CellTracker labeled cells after treatment with 40 µM of the ROCK inhibitor, Y-27632. Results are shown for 5 different experiments with a p-value of <0.0001 using ANOVA followed by the Student-Newman-Keuls multiple comparisons test.

In order to acquire quantitative data on the engulfment process, we employed fluorescence-activated cell sorting (FACS) analysis. In these experiments, GFP positive LIP-expressing cells were mixed in a 1∶3 ratio with control uninfected CellTracker-labeled cells. 48 hrs post-mix cells were collected and analyzed by flow cytometry. We monitored green cells that were also positive for CellTracker label. This population represents the LIP-expressing cells that have engulfed orange-labeled cells. As mentioned above, green LIP-expressing cells also engulf other green cells. However, by mixing the green cells with an excess of orange cells in a 1∶3 ratio, it is more likely that a green cell will encounter an orange cell to engulf rather than another green cell. Nonetheless, the percent of LIP-expressing cells positive for orange label still underrepresents the number of LIP-expressing cells engaged in engulfing other cells. As presented in Figure 5A panel a, nearly 25% of the Ad-LIP MDA-MB-468 cells engulf orange-labeled cells. Meanwhile, only a meager 1.8% of the control AD-GFP MDA-MB-468 cells engulf an orange-labeled cell (Figure 5A panel b). These experiments were repeated 16 different times and we found on average 30% of the LIP-expressing cells engulf CellTracker-labeled cells (Figure 5B). This is significant relative to control GFP only cells with a p-value of <0.0001.

### LIP-mediated Cell Engulfment Requires Rho

Because the time-lapse photography of LIP-mediated cell engulfment shows considerable changes in the actin cytoskeleton occurring, we examined whether Rho signaling, which is a critical regulator of actin, plays a role in the engulfment process. Rho-associated protein kinase (ROCK) proteins, the downstream effectors of Rho GTPases, were inhibited with Y-27632. ROCK inhibition resulted in a significant decrease (p-value: <0.0001) of LIP-mediated cell engulfment as determined by quantitative FACS analysis of green LIP-expressing cells containing CellTracker-labeled cells (Figure 5C). The Rho-ROCK-actin pathway was previously found to be an important regulator during entosis [1].

### LIP-mediated Cell Engulfment is not Dependent on Adenoviral Infection

**Figure 6:**
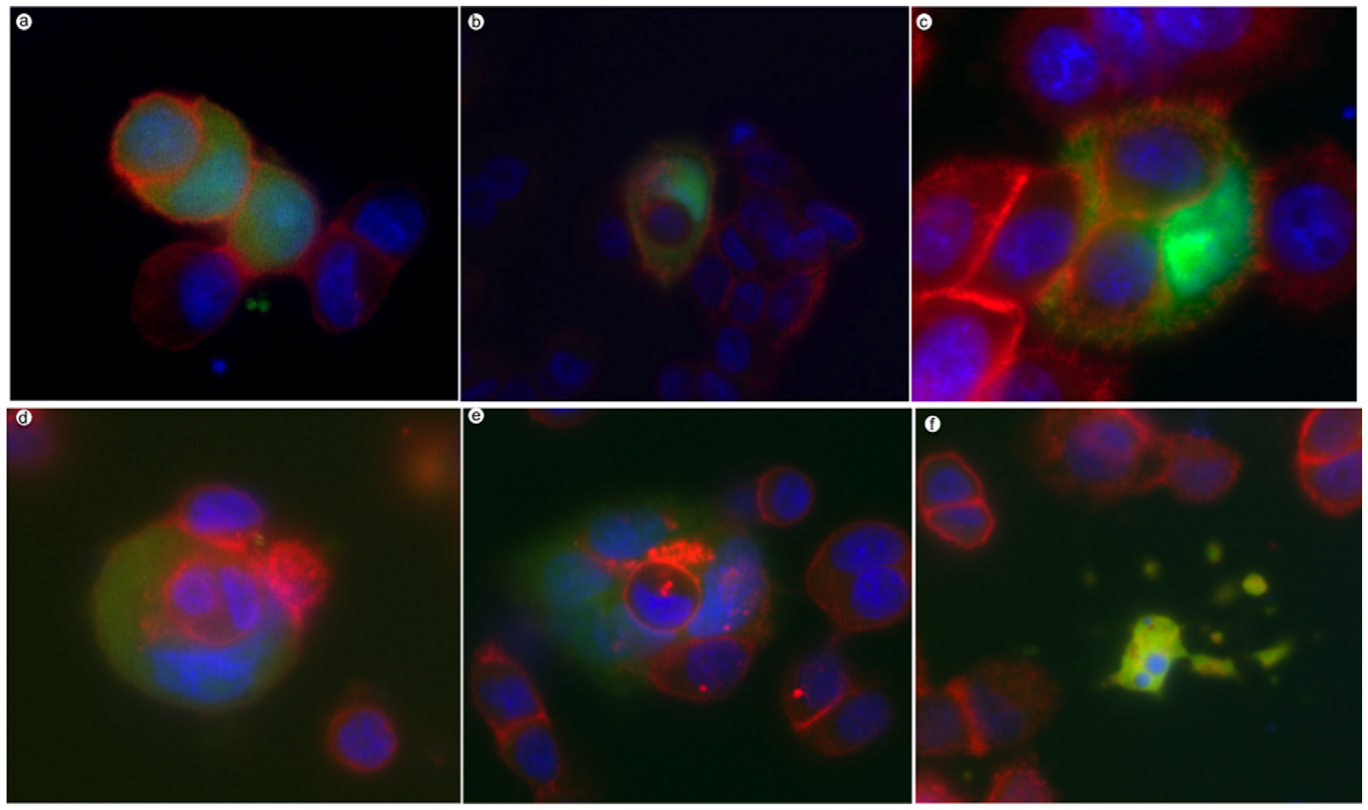
LIP-mediated cell engulfment is not dependent on adenoviral infection. MDA-MB-468 cells cotransfected with LIP and GFP expression vectors were stained with anti-beta-catenin antibody (red) and Hoechst nuclear stain (blue). GFP positive cells in panels a–f are LIP expressing cells. Panels a–f are fluorescent micrographs of separate, randomly chosen fields; cells were imaged 4–6 days post transfection.

**Figure 7:**
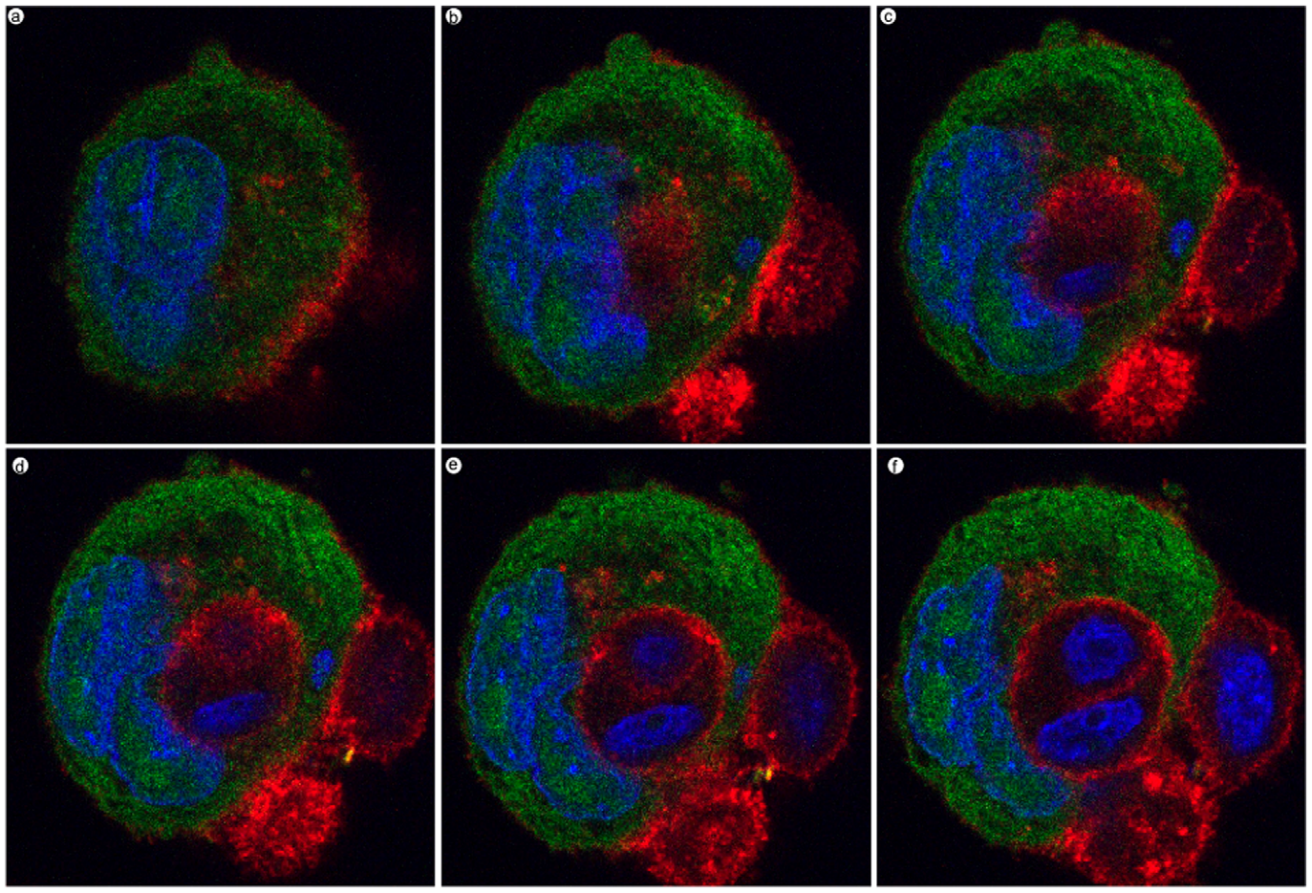
LIP engulfment of neighboring cells via confocal microscopy. LIP-expressing cells stained with anti-beta-catenin antibody (red) and Hoechst nuclear stain (blue) were imaged using confocal microscopy. Images represent a series of Z-stacks.

To address concerns regarding whether adenoviral infection is required for LIP-mediated cell engulfment, we transfected MDA-MB-468 cells with expression vectors for GFP and LIP, or GFP alone. Cells were observed via fluorescent microscopy beginning 48 hrs after transfection. Although the transfection efficiency was low (approximately 5–10%), we were still able to monitor GFP positive cells. At days 4–6 post transfection, cells were fixed and stained for beta-catenin (to better delineate cell boundaries) and nuclear DNA. Similar to our adenoviral studies, we were able to capture engulfing intermediates (Figure 6, panel a) and LIP-expressing cells that have engulfed anywhere from one to three neighboring cells (Figure 6, panels b–e). In addition, we observed LIP-expressing cells that had broken down into vesicles (Figure 6, panel f). Next, we imaged the engulfed cells using confocal microscopy in order to confirm that the LIP-expressing host cell had engulfed its neighboring target cell. Figure 7 depicts a series of images (z-stack) taken of a LIP-expressing cell that has a target cell within (also see Movies S5 and S6). These studies corroborate the fact that it is the overexpression of LIP that leads to this cell engulfment phenomena and not a side effect of using an adenoviral system to overexpress LIP.

## Discussion

We had previously shown that LIP expression in human breast cancer cell lines stimulates autophagy and leads to non-apoptotic cell death [6]. In this report, we present data demonstrating exogenous expression of LIP in the human breast cancer epithelial cell line, MDA-MB-468, leads to the engulfment of neighboring epithelial cells. Cell cycle profiling studies show LIP-expressing cells have a dramatic increase in cells with more than 4 n DNA content. We were able to confirm that the increase in DNA content is due to the engulfment of neighboring cells by using fluorescent CellTracker dyes to monitor this cannibalistic process. Using a quantitative method to monitor cell engulfment, we are able to detect on average approximately 30% of LIP-expressing cells have engulfed a target cell. Engulfment was significantly diminished upon treating with a ROCK inhibitor. When we performed ultrastructure analysis of LIP-expressing cells by TEM, we observe various engulfing intermediates as well as target cells that have been internalized by LIP-expressing host cells. Cell engulfment was observed whether or not adenovirus was employed to overexpress LIP.

When we compare our findings to those published documenting entosis there are some similarities and major differences. Unlike the entosis process, which is described as a cell death mechanism that occurs due to loss of attachment to ECM, our studies were performed with matrix-attached cells. In our studies, we observe LIP cells engulfing the target cells and not an invasion process as described in entosis. Importantly, Overholtzer et al. examined a panel of human cell lines including the MDA-MB-468 human breast cancer cell line for possible entosis. Interestingly, they report that 0% of these cells underwent the entosis process [1]. However, similar to entosis, LIP expression leads to a nonapoptotic cell death process, live cells are engulfed, and this LIP mediated cannibalistic process requires ROCK activity.

Although our studies examined the response to exogenous LIP overexpression, the C/EBPbeta isoform, LIP, is elevated in a variety of processes such as endoplasmic reticulum (ER) stress and oncogenic signaling. ER stress induced by the proteasome inhibitor MG132 was shown to transiently increase C/EBPbeta mRNA levels and notably increased LIP protein levels [10]. Earlier reports by the Hatzoglou group demonstrate increased LIP levels during the late phase of ER stress and this increase in LIP protein level was due to both increased synthesis as well as increased stability of the protein [11]. Whether elevated LIP levels during ER stress could result in cell engulfment has yet to be determined.

Interestingly, there are also reports that couple oncogenic signaling pathways such as Epidermal Growth Factor Receptor (EGFR), Human Epidermal growth factor Receptor 2 (HER2/ErbB2), and (Insulin-like growth factor I Receptor (IGF-IR) to increases in LIP protein levels. Baldwin et al. connect EGFR signaling in mammary epithelial cells to an increase in the LIP levels. They show that C/EBPbeta mRNA levels are unaffected by EGF stimulation and suggest that the increase in LIP expression is controlled posttranscriptionally and is partly due to the interaction of the translational regulatory factor CUG triplet repeat, RNA binding protein 1 (CUGBP1) with C/EBPbeta mRNA [12]. In later studies by Arnal-Estapé et al., they report that hyperactivation of ErbB2 in patient-derived metastatic breast cancer samples leads to an increase in LIP. Similarly, ErbB2 signaling was shown to activate CUGBP1, a downstream effector of ErbB2/PI3K pathway. CUGBP1 appears to favor the production of LIP and inhibition of PI3K activity prevents LIP translation irrespectively of ErbB2 status [13]. More recently, IGF-IR signaling has also been shown to increase LIP expression in an EGFR independent manner in mammary epithelial cells [14]. Li et al. conclude that Akt activity is an important regulator of IGF-IR induced LIP expression and that this elevation in LIP plays a significant role in regulating cellular survival via suppression of anoikis [14]. Cell-in-cell structures have long been noted in tumor samples, and thus it is possible that elevated LIP levels consequent to oncogenic signaling could contribute to their formation by activating cell engulfment.

C/EBPbeta is a well-established regulator of mammary gland development during puberty, pregnancy and involution [15]. Involution of the mammary gland postlactation is a physiological event that involves extensive cell death of the secretory epithelium [16]. Studies have shown that neighboring mammary epithelial cells (MECs) can clear dying MECs in a process termed efferocytosis [16]. In addition to apoptotic death, lysosomal pathways of cell death or autophagy have been implicated to occur during involution [17]. Combining our previous observations that overexpression of LIP induces autophagy and leads to cell death with our current results that LIP overexpression leads to the engulfment of neighboring cells, we considered whether LIP may play a role in involution. However, immunoblot analysis of mouse mammary glands that were isolated at different times throughout pregnancy, lactation, and involution did not show higher levels of LIP during involution. In fact, we were unable to detect LIP protein expression at any of the time points collected (data not shown). Earlier reports by Dearth et al. had similarly concluded that LIP levels are undetectable during gestational proliferation, lactation, or involution in mouse mammary glands [18]. Thus, it is not likely that LIP-mediated cell engulfment plays a role in destruction of the secretory epithelium post weaning. However, one important distinction may be that involution involves the clearance of dying cells, whereas LIP-mediated engulfment characterized here involves live cells.

We did consider whether LIP expression would also stimulate the engulfment of dying cells. To address this question we pretreated CellTracker-violet labeled MDA-MB-468 cells with cisplatin for 8 hrs to induce apoptosis, prior to mixing with green LIP-expressing MDA-MB-468 cells. Using our quantitative cell engulfment assay we did not observe any increase in engulfment of cells undergoing apoptosis; in fact engulfment typically declined (see Figure S4). Given that LIP-overexpression appears to primarily stimulate the engulfment of live cells, it follows that classical don’t-eat-me signals present on viable cells must be in some manner inactivated or overridden. The MDA-MB-468 breast cancer cell line shows very low expression of CD31 and SIRPalpha as ascertained by Affymetrix gene profiling (Tables S1A and S1B) and immunofluorescent staining followed by FACS analysis (Figures S1 and S2). Using these methods, we do observe robust expression of CD47, a don’t-eat-me signal that is a ligand for SIRPalpha, on MDA-MB-468 cells (Figure S3). Recently, Burger et al. have shown that CD47 functions as a molecular switch for erythrocyte phagocytosis. In aging erythrocytes, CD47 undergoes a conformation switch that allows CD47 to bind thrombospondin-1 and act as an “eat me” signal [19]. It is possible that LIP expression could influence the function of CD47, converting it from its classical “don’t eat me” function to an “eat me” signal, but further studies would be necessary to address this possibility.

In regards to the clearance of live cells, studies in Drosophila have recently identified a new tumor-suppression mechanism that eliminates oncogenic imaginal epithelial cells [20]. Mutant epithelial cells that have lost their apicobasal polarity are actually engulfed and eliminated by surrounding normal epithelial cells. Engulfment is dependent on the activation of nonapoptotic JNK signaling in the normal imaginal cells in response to the emergence of neoplastic mutant cells [20]. Given that many components of the Drosophila antitumor cell engulfment pathway are conserved from flies to human, Ohsawa et al speculate that this is an evolutionarily conserved intrinsic tumor-suppression mechanism existing in normal epithelium [20]. Given that LIP can mediate live cell engulfment, it is interesting that C/EBPbeta is a downstream target of JNK signaling [21].

Now that the potential for LIP-mediated engulfment of live cells is known, this may enable the possible recognition of this event during times when LIP levels are elevated, such as ER stress or oncogenic signaling. Like entosis, it remains uncertain whether LIP-mediated engulfment serves a prosurvival role (such as a mechanism to escape immune surveillance) or a growth/tumor suppressor role (such as intrinsic epithelial surveillance and/or via inducing cell death). Specific inhibitors of the LIP-induced engulfment process will be necessary to address these questions. Future studies aimed at characterizing the mechanism by which LIP induces engulfment will facilitate the development of inhibitors and thus a fuller understanding of its biological significance.

## Materials and Methods

### Cell Culture and Adenoviral Constructs

The human breast cancer cell line MDA-MB-468 was obtained from the ATCC (Manassas, VA). MDA-MB-468 cells were maintained in Iscove’s Modified Eagle media supplemented with 10% fetal bovine serum (FBS) from HyClone Laboratories (Logan, UT, USA), 100 U/ml penicillin, 100 µg/ml streptomycin (Life Technologies, Inc.), and 10 µg/ml bovine insulin. Cells were grown at 37°C in a humidified atmosphere containing 5% CO_2_. The adenoviral constructs used in these experiments were previously constructed and described by Duong et al [9]. MDA-MB-468 cells were grown to subconfluency (60–70%) on 100-mm dishes. Cells were either uninfected or adenovirally infected with Ad-GFP or Ad-LIP at a multiplicity of infection (MOI): 5–10 for all experiments.

### Cell Cycle Analysis

DNA cell cycle profiles of sub-confluent (60–70%) cultures were determined by flow cytometry using a BD FACScan (Becton Dickinson, San Jose, CA). Cultures were harvested at 72 hrs post infection by trypsinization and pelleting in the presence of 20% fetal bovine serum at 500 g for 7 min. Cells were then counted using a hemocytometer. Approximately 2×10^6^ cells were washed twice in cold phosphate-buffered saline (PBS) and fixed in ice-cold 70% ethanol overnight. The samples were pelleted at 500 g for 7 min and washed twice with ice-cold PBS. Lastly, the cells were incubated in a staining solution containing 2.5 mg/ml RnaseA, 2.0 mg/ml propidium iodide, 0.1% (v/v) Triton X-100, 1 µM EDTA in 1× PBS for 30–60 min at 4°C in the dark. Data were collected using BD Cellquest software (BD Biosciences Immunocytometry Systems, San Jose, CA), and cell cycle modeling was performed using Modfit software (Verity Software House, Topsham, ME). The cell cycle profile of each population was generated from DNA content data collected from between 20,000 to 30,000 separate events. Doublet exclusion was performed in the analysis of DNA content.

### Cell Internalization Assays

MDA-MB-468 cells were grown to subconfluency (60–70%) on 100-mm dishes. Cells were either uninfected or adenovirally infected with Ad-GFP or Ad-LIP. After 24 hrs, the uninfected monolayer cultures were stained with fluorescent orange or violet CellTracker dyes (Invitrogen, SKU# C2927 or C10094) for 1 hr at 37°C in serum-free media. After 1 hr, cells were replenished with normal growth media for 2–3 hrs. Stained cells were then washed three times with PBS and trypsinized to a single cell suspension in normal growth media. MDA-MB-468 Ad-GFP and Ad-LIP cells were also trypsinized to a single cell suspension and collected in normal growth media. Each population was counted with a hemocytometer and mixed in 1∶3 (LIP or GFP: stained NV cells) ratio. Cells were either plated on 60-mm dishes for FACS analysis or when acquiring images cells were plated on 35-mm dishes fitted with collagen-coated glass coverslips (MatTek Corp, Ashland, MA, USA). In some cases, Hoechst 33342 (Sigma-Aldrich Co., St. Louis, MO) was used to stain DNA. Images presented in Figure 3 were acquired using a Leica DM IRB inverted microscope equipped with a Nikon DXM1200C camera and Metamorph software.

### Electron Microscopy

Control uninfected, Ad-GFP, and Ad-LIP MDA-MB-468 cells were fixed at 48 and 72 hrs post infection with a solution containing 2.5% glutaraldehyde in 0.1 M cacodylate buffer (pH 7.3) for 1 hr at room temperature. Samples were then refrigerated overnight. Postfix, staining, sectioning, and TEM were performed as described [6]. Representative areas were chosen for thin sectioning and viewed with an electron microscope (Philips CM-12 transmission electron microscope).

### Quantitative Cell Engulfment Assay Using Flow Cytometry

In these assays, cells were stained and mixed in 1∶3 as described above. At 24–48 hrs postmixing, cells were trypsinized and collected in Phenol-red free Iscove’s media. Cells were collected for flow cytometry analysis using a 3-laser FACSCanto II instrument, (Becton-Dickinson, San Jose, CA) equipped with standard 5-2-2 filter configuration. FACSDiVa Software was used for data acquisition and analysis. Inhibition of ROCK was performed with 20–40 µM of Y-27632 (EMD, Calbiochem, product number 688000) for 36–48 hrs postmix until cell populations were prepared for FACS analysis. Apoptosis was induced by treatment with 50 µM cisplatin for 8 hrs prior to mixing. The induction of apoptosis was confirmed by immunoblotting for activated (cleaved) caspase 3 in the treated cells.

### Transfections

2×10^5^ MDA-MB-468 cells were plated in 35-mm dishes fitted with collagen-coated glass coverslips (MatTek Corp, Ashland, MA, USA) and transfected in complete Iscove’s growth medium with *Trans*IT-LT1 transfection reagent (Mirus Bio LLC, Madison, WI) using 5 µg of pLZRS-IRES-GFP DNA or 4 µg pLZRS-IRES-GFP DNA and 1 µg of pcDNA3.1hisLIP at a 1∶1 ratio of *Trans*IT-LT1 reagent:DNA according to manufacturer’s instructions. Cells were imaged at 4 or 6 days post transfection.

### Indirect Immunostaining and Image Acquisition

Transfected cells were prepared for immunofluorescence studies as described previously [6]. Briefly, MDA-MB-468 cell cultures were washed three times in PBS, fixed in 3.7% formalin in PBS for 30 min at room temperature, washed an additional three times, and processed for indirect immunofluorescence. Cells were permeabilized in PBS containing 0.1% Triton X-100 for 20 min at room temperature. The cells were washed and nonspecific binding sites were blocked in PBS containing 5% BSA (Fraction V, Sigma) at 4°C for 24 hrs. Immediately following aspiration of the blocking solution, the cells were incubated with beta-catenin polyclonal antibody (Sigma, C2206) at a dilution of 1∶1,000 in PBS containing 2% BSA and 0.1% Triton X-100 for 2 hrs at room temperature. The cells were washed as described above. Cells were then incubated for an additional hour at room temperature in the dark with fluorescent-conjugated Alexa 594 goat-anti-rabbit secondary antibody diluted to a final concentration of 2 µg/ml in PBS containing 2% BSA and 0.1% Triton X-100. The cells were then washed three times in PBS containing 0.1% Triton X-100 and a few final rinses with double-distilled water. The DNA stain, Hoechst 33342 (Sigma-Aldrich Co., St. Louis, MO), was used to label the nucleus. Images were acquired with an Eclipse TE2000-E wide-field fluorescent microscope (Nikon) equipped with a 60×, 1.4NA, oil immersion lens and a cooled charge-coupled device (CCD) camera by the use of Metamorph software.

### Time-Lapse Microscopy

Cells were mixed and plated on coverglass bottom dishes (MatTek, Ashland, MA) as described above. At 36 hrs postmix images were acquired automatically at multiple locations on the coverslip using a Zeiss LSM 510 inverted confocal microscope fitted with a 20× objective. The microscope was housed in a custom-designed 37°C chamber with a secondary internal chamber that delivered humidified 5% CO_2_. For Movies S1 and S2, fluorescence images were obtained every 4 min for a period of 12–14 hrs using Zeiss image processing software (LSM 5; Carl Zeiss). Imaging conditions did not adversely affect cell proliferation or viability. Movies S3 and S4 were imaged using an Olympus BX61WI upright microscope fitted with a 40× objective. The microscope was housed in a custom-designed 37°C chamber and Volocity (Perkin Elmer) software was used for image acquisition.

### Confocal Microscopy

To confirm if target cells were completely internalized by host LIP-expressing cells, cells were imaged using a confocal laser-scanning microscope (model LSM 510; Carl Zeiss, Inc.) using a 63× objective. Optical section series were collected with a spacing of 0.4µm in the z-axis through ∼3µm thickness of the cell-in-cell complex. The images from triple labeling were simultaneously collected using a dichroic filter set with Zeiss image processing software (LSM 5; Carl Zeiss). Digital data were exported into Adobe Photoshop for presentation and as quick-time movie.

### Immunostaining for Cell Surface “Don’t eat me” Signals and FACS Analysis

MDA-MB-468 cells were grown to subconfluency (60–70%) on 100-mm dishes. Cells were either uninfected or adenovirally infected with Ad-GFP or Ad-LIP. Cells were then plated in 60-mm dishes and collected at 48–72 hrs post infection to process for flow cytometric analysis. HUVECs (a gift from J. Chen, Dept. of Cancer Biology,Vanderbilt Univ.) used as a positive control for CD31 cell surface expression were processed in a similar manner as MDA-MB-468 cells. Cells were trypsinized and collected in normal growth media to perform cell counts. Cells were then washed with FACS buffer (0.5% BSA, 2 mM EDTA in PBS) and a 100 µl cell suspension was incubated with antiCD31-APC (eBioscience cat. # 17-0319-41), antiCD47-APC (eBioscience cat. # 17-0479-41), or antiSIRPa-APC (eBioscience cat. # 17-1729-41) according to the manufacturer’s protocol. Cells were stained in the dark for at least 30 min at 4°C. Following incubation, cells were collected by centrifugation, washed 3 times in FACS buffer and analyzed by flow cytometry. Cell surface expression of CD31, CD47, and SIRPa was monitored by flow cytometry using a 3-laser FACSCanto II instrument, (Becton-Dickinson, San Jose, CA) equipped with standard 5-2-2 filter configuration. FACSDiVa Software was used for data acquisition and analysis.

### Statistical Analysis

Quantitative data are expressed as means. For comparisons between two groups paired t-test and Wilcoxon matched pairs test was used. In order to compare multiple groups, ANOVA followed by the Friedman test or the Student-Newman-Keuls multiple comparisons test was used. Prism 5.0 (GraphPad, La Jolla, CA) was used for all analyses.

## Supporting Information

Figure S1 **CD31 cell surface expression is very low in MDA-MB-468 cells.** Quantitative FACS analysis of CD31 cell surface expression in control no virus (NV) (top left panel), Ad-GFP (top right panel), and Ad-LIP (bottom right panel) MDA-MB-468 cells are shown. Positive control HUVECs are shown in bottom left panel. Percent of cells positive for CD31 cell surface expression is presented for each population. Representative dot plots are shown; experiments were repeated three separate times at 48 hrs post infection.
(TIF)

Figure S2 **SIRPa cell surface expression is very low in MDA-MB-468 cells.** Quantitative FACS analysis of SIRPa cell surface expression in control no virus (NV) (top panel), Ad-GFP (middle panel), and Ad-LIP (bottom panel) MDA-MB-468 cells are shown. Percent of cells positive for SIRPa cell surface expression is presented for each population. Representative dot plots are shown; experiments were repeated three separate times at 48 hrs post infection.
(TIF)

Figure S3 **Characterization of CD47 cell surface expression in MDA-MB-468 cells.** Quantitative FACS analysis of CD47 cell surface expression in control no virus (NV) (top panels), Ad-GFP (middle panels), and Ad-LIP (bottom panels) MDA-MB-468 cells are shown. Representative dot plots and flow cytometric histograms with mean fluorescence intensity for each population are presented. Experiments were repeated three separate times at 48 hrs post infection.
(TIF)

Figure S4 **Apoptotic cells are not targeted for engulfment by LIP-expressing MDA-MB-468 cells.** Quantification of the percent of GFP positive cells (Ad-LIP or control Ad-GFP infected) that have engulfed either uninfected CellTracker violet-labeled MDA-MB-468 cells treated with 50 µM cisplatin for 8 hrs prior to mixing to induce apoptosis (gray bars) or control, untreated, uninfected CellTracker violet-labeled MDA-MB-468 cells (black bars). FACS analysis was performed 48 hrs after mixing the two fluorescently labeled cell populations.
(TIF)

Table S1 **A.** **Expression of “don’t eat me” signals in MDA-MB-231 breast cancer cells.** MDA-MB-231 breast cancer cells infected with AdLIP, Ad-GFP or uninfected cells were harvested 72 hrs post infection and total RNA was subject to genomic profiling using Affymetrix human U133Plus 2.0 microarrays. Expression values for the indicated probe sets are shown and are representative of three independent determinations performed by the Vanderbilt Microarray Shared Resource. **B. Expression of “don’t eat me” signals in MDA-MB-468 breast cancer cells.** MDA-MB-468 breast cancer cells infected with AdLIP, Ad-GFP or uninfected cells were harvested 72 hrs post infection and total RNA was subject to genomic profiling performed by the Vanderbilt Microarray Shared Resource using Affymetrix human U133Plus 2.0 microarrays. Expression values for the indicated probe sets are shown.
(PDF)

Movie S1 **LIP-expressing MDA-MB-468 engulfs neighboring cell.** Time-lapse analysis of matrix-attached GFP positive LIP-expressing MDA-MB-468 cells as it wraps its membrane and engulfs neighboring CellTracker orange-labeled MDA-MB-468 cell. Images were acquired every 4 min for 12 hrs.
(MOV)

Movie S2 **LIP-expressing MDA-MB-468 cells are very active.** Time-lapse analysis of LIP-expressing cells that have engulfed CellTracker orange-labeled cells and begin to breakdown into smaller vesicles. Images were acquired every 4 min for 12 hrs.
(MOV)

Movie S3 **LIP-expressing MDA-MB-468 cells can release engulfed cell**. Time-lapse analysis shows a GFP positive LIP-expressing cell open and expel an engulfed labeled-cell. Images were acquired every 4 min for 6 hrs.
(MOV)

Movie S4 **LIP-expressing MDA-MB-468 cells breakdown and take engulfed cells.** Time-lapse analysis of a GFP positive LIP-expressing that has two CellTracker labeled cells.
(MOV)

Movie S5 **LIP-mediated engulfment of neighboring cell via confocal microscopy.** Z series of confocal microscopic analysis representative of a LIP-expressing cell that has engulfed a neighboring cell.
(MOV)

Movie S6 **LIP-mediated engulfment of neighboring cell via confocal microscopy.** Three-dimensional reconstruction along the Z-axis depicting a LIP-expressing cell that has engulfed a neighboring cell.
(MOV)
