# Supplementary figures and images for: Cells Expressing the C/EBPbeta Isoform, LIP, Engulf Their Neighbors
Source: PLoS One. 2012 Jul 31;7(7):e41807. doi: 10.1371/journal.pone.0041807 (PMC3409234; doi:10.1371/journal.pone.0041807)

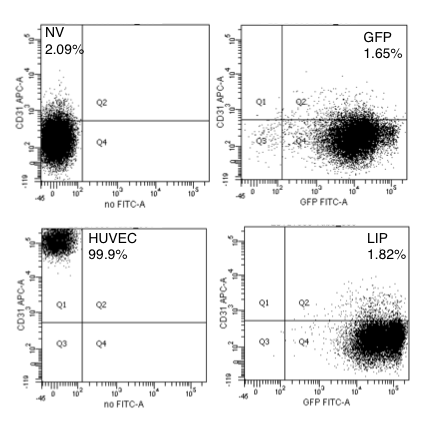

Supplement: Figure S1 — CD31 cell surface expression is very low in MDA-MB-468 cells. Quantitative FACS analysis of CD31 cell surface expression in control no virus (NV) (top left panel), Ad-GFP (top right panel), and Ad-LIP (bottom right panel) MDA-MB-468 cells are shown. Positive control HUVECs are shown in bottom left panel. Percent of cells positive for CD31 cell surface expression is presented for each population. Representative dot plots are shown; experiments were repeated three separate times at 48 hrs post infection. (TIF) [file pone.0041807.s001.tif]

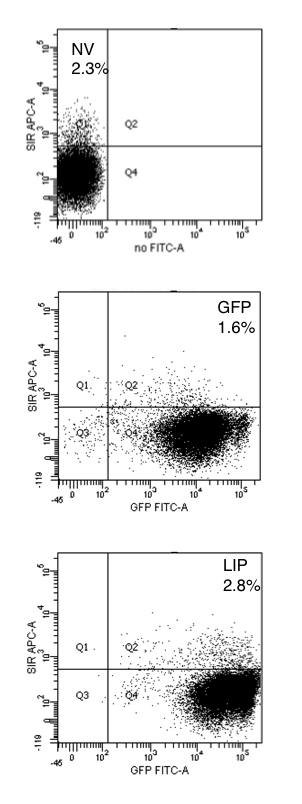

Supplement: Figure S2 — SIRPa cell surface expression is very low in MDA-MB-468 cells. Quantitative FACS analysis of SIRPa cell surface expression in control no virus (NV) (top panel), Ad-GFP (middle panel), and Ad-LIP (bottom panel) MDA-MB-468 cells are shown. Percent of cells positive for SIRPa cell surface expression is presented for each population. Representative dot plots are shown; experiments were repeated three separate times at 48 hrs post infection. (TIF) [file pone.0041807.s002.tif]

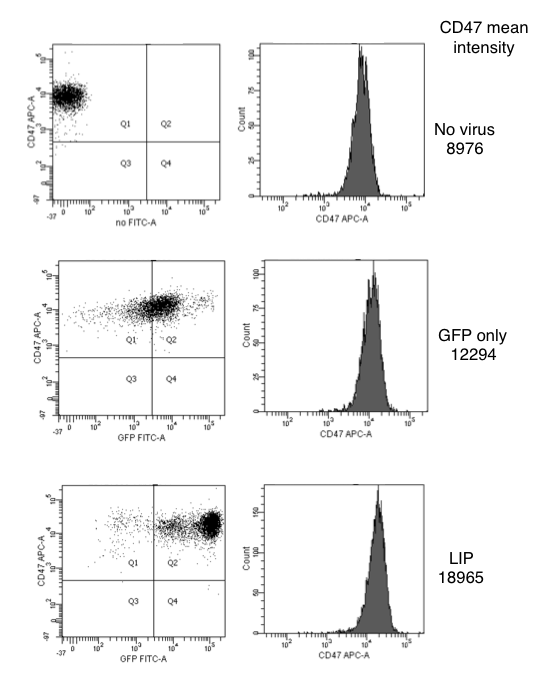

Supplement: Figure S3 — Characterization of CD47 cell surface expression in MDA-MB-468 cells. Quantitative FACS analysis of CD47 cell surface expression in control no virus (NV) (top panels), Ad-GFP (middle panels), and Ad-LIP (bottom panels) MDA-MB-468 cells are shown. Representative dot plots and flow cytometric histograms with mean fluorescence intensity for each population are presented. Experiments were repeated three separate times at 48 hrs post infection. (TIF) [file pone.0041807.s003.tif]

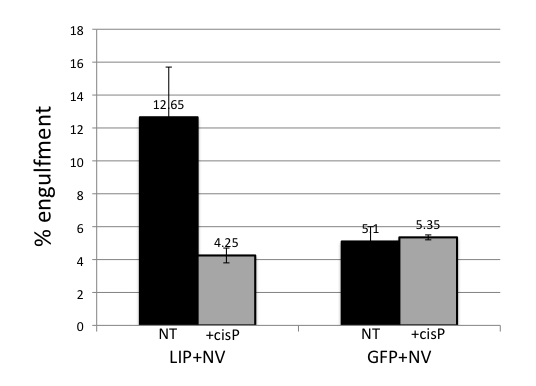

Supplement: Figure S4 — Apoptotic cells are not targeted for engulfment by LIP-expressing MDA-MB-468 cells. Quantification of the percent of GFP positive cells (Ad-LIP or control Ad-GFP infected) that have engulfed either uninfected CellTracker violet-labeled MDA-MB-468 cells treated with 50 µM cisplatin for 8 hrs prior to mixing to induce apoptosis (gray bars) or control, untreated, uninfected CellTracker violet-labeled MDA-MB-468 cells (black bars). FACS analysis was performed 48 hrs after mixing the two fluorescently labeled cell populations. (TIF) [file pone.0041807.s004.tif]
