# Supplementary material for: Cells Expressing the C/EBPbeta Isoform, LIP, Engulf Their Neighbors
Source: PLoS One. 2012 Jul 31;7(7):e41807. doi: 10.1371/journal.pone.0041807 (PMC3409234; doi:10.1371/journal.pone.0041807)
Supplement: Table S1 — A. Expression of “don’t eat me” signals in MDA-MB-231 breast cancer cells. MDA-MB-231 breast cancer cells infected with AdLIP, Ad-GFP or uninfected cells were harvested 72 hrs post infection and total RNA was subject to genomic profiling using Affymetrix human U133Plus 2.0 microarrays. Expression values for the indicated probe sets are shown and are representative of three independent determinations performed by the Vanderbilt Microarray Shared Resource. B. Expression of “don’t eat me” signals in MDA-MB-468 breast cancer cells. MDA-MB-468 breast cancer cells infected with AdLIP, Ad-GFP or uninfected cells were harvested 72 hrs post infection and total RNA was subject to genomic profiling performed by the Vanderbilt Microarray Shared Resource using Affymetrix human U133Plus 2.0 microarrays. Expression values for the indicated probe sets are shown. (PDF) [file pone.0041807.s005.pdf]

Supplementary Table 1A. Expression of "eat me" signals in MDA-MB-231 breast cancer cells. MDA-MB-231 breast cancer cells infected with AdLIP, Ad-GFP or uninfected cells were harvested 72 hrs postinfection and total RNA was subject to genomic profiling using Affymetrix human U133Plus 2.0 microarrays. Expression values for the indicated probe sets are shown and are representative of three independent determinations performed by the Vanderbilt Microarray Shared Resource.

Gene	Probe ID	231-No virus	231-GFP	231-LIP
CD31 (PECAM1)	208982_ _at	108	92	87.4
	1559921_at	139	94	118
	208981_at	86	87	58
	208983_s_at	2	37	9
SIRPalpha	217240_at	80	15	49
	202895_s_at	13	80	2
	202896_s_at	65	53	45
	202897_s_at	10	3	9
CD47	213055_at	141	193	100
	213856_at	1	4	4
	213857_s_at	10684	10869	11985

Supplementary Table 1B. Expression of "eat me" signals in MDA-MB-468 breast cancer cells. MDA-MB-468 breast cancer cells infected with AdLIP, Ad-GFP or uninfected cells were harvested 72 hrs postinfection and total RNA was subject to genomic profiling performed by the Vanderbilt Microarray Shared Resource using Affymetrix human U133Plus 2.0 microarrays. Expression values for the indicated probe sets are shown.

Gene	Probe ID	468-No virus	468-GFP	468-LIP
CD31 (PECAM1)	208982_ _at	64	69	47
	1559921_at	43	46	44
	208981_at	25	39	23
	208183_s_at	7	8	7
SIRPalpha	217240_at	28	32	30
	202895_s_at	11	15	12
	202896_s_at	86	86	99
	202897_s_at	36	44	41
CD47	213055_at	60	56	59
	213856_at	9	11	15
	213857_s_at	2111	2050	2560
